# Salient Points to Observe in Panfacial Fracture Management

**DOI:** 10.5812/traumamon.8090

**Published:** 2012-10-10

**Authors:** Ali Hassani, Mohammad Hosein Kalantar Motamedi

**Affiliations:** 1 Department of Oral and Maxillofacial Surgery, Azad Islamic University of Medical Sciences, Dental Branch, Tehran, IR Iran; 2 Trauma Research Center, Baqiyatallah University of Medical Sciences, Tehran, IR Iran

**Keywords:** Panfacial, Fracture, Management

Dear Editor,

Treatment of facial trauma, damage to the dentition and anatomic structures subsequent to maxillofacial injury is an issue of paramount importance in traumatology. Because in this field, unlike other parts of the body, not only does the surgeon have to deal with the management of the facial fractures, but must also restore the facial functions and features such as visual function (i.e. diplopia), olfaction, breathing (i.e. airway management), mastication (i.e. restoration of teeth and occlusion), deglutition and articulation (in addition to the facial appearance of the patient and symmetry). In no other part of the body is the management of trauma so complex. In patients with multiple fractures of the upper, lower and midface are generally referred to as panfacial fractures treatment is extremely complicated. Often, such fractures are associated with neurological deficits, and require ICU care for other multiple traumas. The quality of life, ability return to work and management of PTSD are other fundamental issues inherent to trauma care, rehabilitation and counseling which are of tantamount importance and must not be neglected (1).

**Figure 1:**
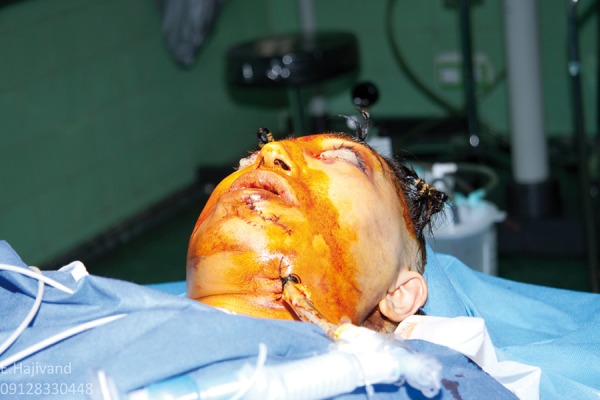
Submandibular Intubation in a Patient With Panfacial Fractures

**Figure 2:**
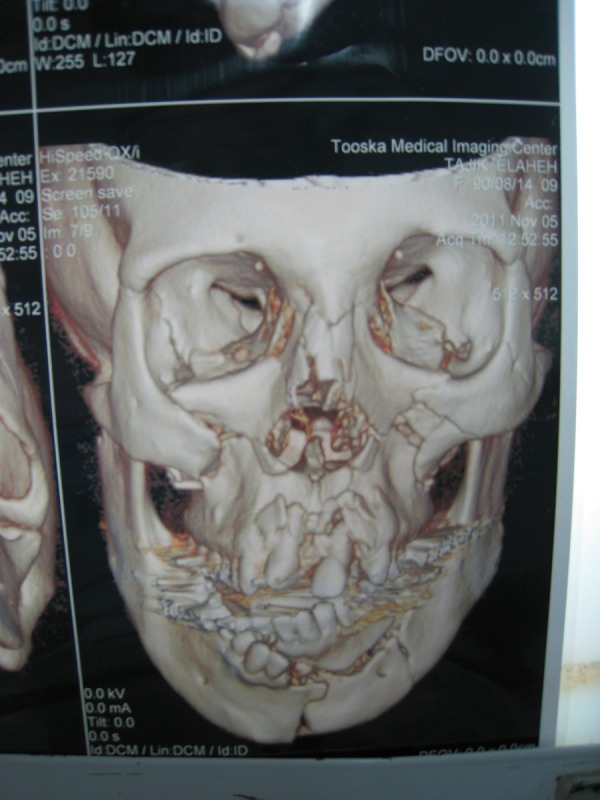
3D reconstruction CT of a Patient With Panfacial Fractures. Note Nasoethmoidorbital Fracture, LeFort 1 and 2, Zygoma, Maxilla, Alveolar, Mandibular and Dental Fractures

Nonetheless, the following listed points are worthy of mentioning when faced with these patients and may be of interest for your readership:

1. Complete and exact assessment of not only facial injuries but also, concomitant bodily injuries which may not be evident is necessary. Admission of patients from the emergency ward to maxillofacial ward must be done only after consultations are complete and the patient has been cleared from the other wards (i.e. neurosurgery, surgery, internal medicine etc)(2).

2. Closure of open wounds of the face and oral mucosa and avulsed teeth in patients whose surgery is to be delayed (temporary treatment).

3. Attention to and provision of oral hygiene, and nutrition especially in ICU patients (i.e. in a coma).

4. Preoperative photographs, radiographs and CT scans are mandatory (Figure 1) (1).

5. Consultation, coordination and cooperation with other relevant departments (such as neurosurgery, ophthalmology, otolaryngology, anesthesiology etc.) is prudent (3).

6. Use of submental or Altemeier intubation procedure to obviate the need for tracheotomy and preventing changing of the intubation tube from nasal to oral (1) (Figure 2).

7. Attempt to treat fractures and reconstruction of the face in one surgical operation (delayed and secondary damages of face and jaws, soft tissue, orbit, canthal ligament, nasoethmoidofrontal complex, condyle and occlusion is complicated following scarring, contraction of soft tissues and muscles, malunion and or callous formation)(4).

8. Endoscope availability both for intubation and for examining the maxillary sinus and inferior floor of the orbit and ethmoidofrontal sinus. May need submental intubation (Figure 1).

9. Use of appropriate incision and flaps for exposure of segments (priority given to use of available lacerations) (5).

10. Attention to procedures for rigid fixation of fractures (reconstruction and exact fixation of the zygomatic arch for preserving projection and correct fixation of the mandibular condyle if needed to restore the vertical dimension of the face with multiple fractures, Figure 2)(4).

11. Attention to simultaneous reconstruction (bone graft or alloplasts) (5).

12. Resuspension of facial soft tissue
